# Medical Debt and Hunger-Coping Economic Tradeoffs Among Charitable Food Assistance Clients

**DOI:** 10.3390/nu18213475

**Published:** 2026-10-22

**Authors:** Maha Almohamad, Crystal Cartwright, Fred P. Brooks, Craig Gundersen, Daphne C. Hernandez

**Affiliations:** 1 Department of Epidemiology, Center for Healthy Communities, The University of Texas Health Science Center at Houston, 1200 Pressler St., Houston, TX 77030, USA; maha.almohamad@uth.tmc.edu; 2 Cizik School of Nursing, The University of Texas Health Science Center at Houston, 6901 Bertner Ave., Suite 591, Houston, TX 77030, USA; crystal.cartwright@uth.tmc.edu; 3 School of Social Work, Andrew Young School of Policy Studies, Georgia State University, 55 Park Place NE, Atlanta, GA 30303, USA; fbrooks2@gsu.edu; 4 Hankamer School of Business, Baylor University, 1621 S. 3rd St., Waco, TX 76706, USA; craig_gundersen@baylor.edu

**Keywords:** economic hardship, financial hardship, food insecurity, material hardship

## Abstract

**Objective**: Medical debt is a long-term hardship, typically unplanned, and difficult to resolve. Hunger-coping economic tradeoffs (e.g., choosing between food or another basic need) are smaller financial constraints related to implementing short-term strategies to resolve cash shortages. This study examined associations between long-term financial constraint (medical debt) and engagement in short-term financial strategies (hunger-coping economic tradeoffs) among charitable food assistance (CFA) recipients. **Methods:** Survey data were collected in 10 Houston-area and 10 Atlanta-area food pantries in 2022. Hunger-coping economic tradeoff experiences were based on affirmative responses to whether anyone in the household ever had to choose between food and five basic needs. Covariate-adjusted logistic regression models were used to assess the relationship between medical debt (defined as unpaid medical bills) and each hunger-coping tradeoff. **Results**: Among 999 participants (mean age, 55 years), 41% reported medical debt. Participants with medical debt had greater odds of having to choose between paying for food or (1) childcare (AOR = 1.92, 95% CI 1.37–2.69, *p* < 0.001), (2) utilities (AOR = 2.05, 95% CI 1.59–2.64, *p* < 0.001), (3) rent/mortgage (AOR = 2.34, 95% CI 1.75–3.14, *p* < 0.001), (4) transportation/gas (AOR = 2.81, 95% CI 2.05–3.85, *p* < 0.001), and (5) education (AOR = 2.18, 95% CI 1.42–3.34, *p* < 0.001). **Conclusions:** Long-term hardship (i.e., medical debt) was associated with CFA recipients reporting engaging in short-term financial strategies (i.e., hunger-coping economic tradeoffs) to make ends meet. These findings highlight the need to screen CFA recipients for medical debt, economic hardship, and financial management skills, with food pantries serving as key referral points.

## 1. Introduction

Medical debt is unpaid medical expenses and is correlated with financial distress [1]. In the United States, 36% of households have medical debt [2], and lower-income households, Black and Hispanic adults, women, and individuals who are uninsured are more likely to have medical debt [3]. Medical debt can delay or preclude payment for other necessities, such as food and utilities [4,5]. Consequently, the financial strain of meeting basic needs may help explain why some U.S. adults perceive that they may never be able to pay off medical debt [3].

In light of food price inflation [6,7], as the cost of food has increased, so has the number of individuals depending on charitable food assistance (CFA) since 2019 [8]. Prior work has documented that CFA clients often face material hardship, such as foregoing rent, utility payments, or health care, to preserve food access. The emerging literature on the topic of hunger-coping economic tradeoffs, such as choosing between purchasing food or another basic need (e.g., transportation), indicates tradeoffs are associated with heightened food insecurity and food assistance program participation [9,10,11]. The extent to which medical debt contributes to such tradeoffs, especially among CFA clients, has not been thoroughly explored.

Understanding how medical debt may shape hunger-coping economic tradeoffs requires a framework that distinguishes between persistent financial constraints and immediate financial pressures. The time horizon theoretical model [12] conceptualizes hardship along two dimensions: long-term solutions to financial constraint, and short-term solutions to financial constraint. This two-factor model suggests that hardships can be categorized according to both the duration of the hardship and the magnitude of the financial constraint. Within this framework, medical debt represents a long-term hardship because it is typically unplanned, persists across multiple time periods, involves owing a large dollar amount, and is more difficult to resolve. As such, medical debt may be characterized as a financial crisis. In contrast, basic needs such as food and utilities are usually included in monthly budgets, but temporary cash shortages can force households to make hunger-coping economic tradeoffs. Hunger-coping economic tradeoffs are generally smaller financial constraints and are related to short-term strategies that include reallocating money from food to pay a bill (e.g., utility bill), missing a payment, or eating less until the next pay cycle. Once the immediate financial shortfall is resolved (e.g., utility bill is paid), the tradeoff is removed and the burden is lessened.

This study advances understanding of the relationship between long-term financial hardship and short-term coping behaviors by examining whether medical debt is associated with hunger-coping economic tradeoffs among CFA recipients. We hypothesized that medical debt heightens the odds of engagement in five hunger-coping economic tradeoffs. Thus, long-term debt may reduce a household’s daily budget, requiring households to engage in short-term strategies to address basic needs. This study has the potential to demonstrate that medical debt may function not only as a healthcare financial burden but also as a potential upstream determinant of food-related economic tradeoffs and material hardship. Findings could inform interventions aimed at reducing both medical debt and economic tradeoffs related to food and other basic needs.

## 2. Methods

### 2.1. Participants

The data were from a larger study focused on food pantry usage among CFA recipients. Participants were recruited in 2022 (March–August) through in-person outreach and printed flyers at 10 food pantry sites in Atlanta and 10 in Houston. Data were collected in person during regular weekday operating hours. Individuals were eligible to participate if they were (1) at least 18 years old, (2) able to speak English or Spanish, and (3) food pantry clients with an active profile in the food pantry attendance database. Participants received a $25 gift card for survey completion, and all participants completed their survey in one visit. Informed consent was obtained from all participants, and the study was approved by the Institutional Review Board of Georgia State University and The University of Texas Health Science Center at Houston.

Of the 1435 participants who completed the survey, 999 had complete data on all the variables of interest for this study. The highest proportions of missing data were for household size (45%) and income (33%). The proportions of missing data were low for both independent (11%) and dependent (2%) variables, and the remaining missing data (9%) were derived from other covariates. Compared to the participants with complete data (N = 999), those with incomplete data (n = 436) tended to be older, Black, high school graduates, unemployed, and from Atlanta, with an annual income ≤$10,000 and more people in the household. Compared to participants with incomplete data, those with complete data were more likely to be Hispanic and speak Spanish and more likely to experience hunger-coping economic tradeoffs in relation to childcare, rent/mortgage, and education.

### 2.2. Measures

Medical Debt. Participants were asked whether they or anyone in their household currently had unpaid medical or hospital bills. Participants who answered “yes” were classified as having household medical debt, whereas those who answered “no” were classified as not having household medical debt.

Hunger-Coping Economic Tradeoffs. Participants were asked five separate questions regarding whether anyone in their household had ever had to choose between paying for food and paying for another basic need: (1) childcare, (2) utilities, (3) rent/mortgage, (4) transportation/gas, and (5) education (e.g., tuition) [13]. For each item, responses were coded dichotomously (1 = yes, 0 = no). Five separate outcome variables were created, with an affirmative response indicating that the household had experienced that specific hunger-coping economic tradeoff.

Covariates. Sociodemographic characteristics were included in all regression models as covariates to control for factors related to medical debt and hunger-coping economic tradeoffs: age (continuous, years), sex (male [reference] vs. female), race/ethnicity (Black [reference], Hispanic, White, Other Race), marital status (married vs. single [reference]), education (less than high school diploma, high school diploma, more than a high school [reference]), annual household income ($0–$10,000 [reference], $10,001–$15,000, $15,001–$25,000, and ≥$25,001), employment status (full-time, part-time, currently not employed [reference]), health insurance (private [reference], Medicare, uninsured, and Medicaid), participation in the Supplemental Nutrition Assistance Program (SNAP; yes vs. no [reference]), household size (continuous, number of household members), language in which the survey was administered (Spanish vs. English [reference]), and the city in which data was collected (Atlanta vs. Houston [reference]). Categorical variables were entered into the regression models using indicator variables, with the specified reference categories serving as the comparison group.

### 2.3. Analytical Strategy

Descriptive analyses were used to summarize the data from the full sample, and bivariate analyses were conducted by the medical debt category. T-tests were used to test for differences among continuous variables, and chi-squared tests were used for categorical variables. A sequence of logistic regression models was conducted to evaluate the association between medical debt and the five hunger-coping economic tradeoffs. All models controlled for sociodemographic covariates. Standard errors in all regression models were corrected to account for multiple observations within a food pantry. All analyses were conducted using STATA software, version 16 [14].

## 3. Results

### 3.1. Sample Characteristics and Descriptive Statistics

Across the analytic sample (N = 999; mean age = 55.41, standard deviation = 14.30), 41% of participants reported medical debt. Hunger-coping economic tradeoffs were widespread with a greater proportion of the participants reporting choosing between food and utilities (47%), between food and transportation/gas (42%), and between food and rent/mortgage (41%) (Table 1).

Participants were predominantly female (77%), Hispanic (51%), single (52%), high school educated or more (65%), and employed (51%). About three-quarters had an annual household income below $25,000, 40% were enrolled in Medicaid, and 30% participated in SNAP. The average household size was 3.7 people. Over one-third of participants completed the survey in Spanish, and approximately two-thirds resided in Houston (Table 1).

Bivariate analyses showed that compared to participants without debt, those with medical debt were more likely to report tradeoffs across all five domains: childcare (18% vs. 11%, *p* < 0.01), utilities (57% vs. 40%, *p* < 0.001), rent/mortgage (51% vs. 34%, *p* < 0.001), transportation (57% vs. 32%, *p* < 0.001), and education (20% vs. 10%, *p* < 0.001) (Table 1). Compared to participants without medical debt, those with medical debt were more likely to be Black or White participants, have more than a high school education, have private insurance, reside in Atlanta, and have more household members. Participants without medical debt were more likely to be Hispanic, speak Spanish, have less than a high school education, and report annual household incomes of $10,000 or less.

### 3.2. Associations Between Medical Debt and Hunger-Coping Economic Tradeoffs

Adjusted logistic regression results indicated a consistent pattern across all five outcomes (Table 2). Participants with medical debt had approximately two- to threefold greater odds of reporting hunger-coping economic tradeoffs than those without medical debt. The association was strongest for having to choose between paying for food and transportation/gas (adjusted odds ratio [AOR] = 2.81, 95% confidence interval [CI] 2.05–3.85, *p* < 0.001), and weakest for having to choose between paying for food and childcare (AOR = 1.92, 95% CI 1.37–2.69, *p* < 0.001). All associations were statistically significant (*p* < 0.001). 

## 4. Discussion

Guided by the time horizon model [12], this study evaluated the association between medical debt and five hunger-coping economic tradeoffs among a sample of CFA recipients. Individuals with medical debt were significantly more likely to make tradeoffs between food and essential expenses, including childcare, utilities, rent/mortgage payments, transportation, and education. These results are consistent with prior studies showing that households burdened by medical debt often face broader material hardships, such as challenges paying for rent, utilities, and food [4,5]. They also align with the hunger-coping tradeoff literature, which suggests that food assistance, while important, may be insufficient to address the broader financial challenges facing economically vulnerable families [9,10,11].

These findings further suggest that the effects of medical debt extend beyond healthcare finances by constraining household resources. For households relying on CFA or other forms of food assistance, medical debt may exacerbate existing economic pressure and increase the likelihood of engaging in short-term strategies to manage competing basic needs. The economic pressure may also contribute to healthcare avoidance, particularly among individuals with low income and chronic health conditions who are already vulnerable to missed or deferred healthcare [15,16]. Deferring healthcare reduces exposure to new medical expenses and, consequently, the risk of accumulating additional medical debt, which could explain why the poorest adults in our sample were less likely to report medical debt.

The results have potential implications for service interventions within CFA. Although not all users of CFA are food insecure, financial management confidence and specific financial management skills can reduce the risk of food insecurity in this population [17,18]. Partnering with healthcare providers and integrating financial literacy courses or debt relief and counseling services [19,20] into pantry settings may help clients manage both short- and long-term financial hardships [21]. Future studies should explore the integration of wraparound services at food pantries to screen for medical debt, financial hardship, or SNAP eligibility and to help identify households that might benefit from additional resources.

Although this study provides important insights, several limitations should be considered. The sample consisted of adults accessing food pantries. This is consistent with the prior literature on hunger-coping economic tradeoffs that has focused on populations that access food pantries and food banks [9,10,11]. Thus, the results are biased towards a population that may experience elevated levels of financial and material hardship. There are other interconnected factors that the study did not directly account for, such as healthcare needs, that could influence the association between medical debt and economic tradeoffs. Thus, medical debt is a potential contributor to experiencing hunger-coping economic tradeoffs, but not the sole indicator.

In addition, medical debt was measured broadly, which limited our ability to explore the magnitude and duration of debt, the broader financial context of other long-term debt obligations, and the specific healthcare services associated with the reported debt (e.g., preventive care, primary care, chronic disease care, catastrophic care). Because our data did not permit an analysis of the amount and duration of medical debt, we could not quantify the medical debt measure as a long-term financial constraint; however, previously published research suggests medical debt is a long-term debt [22]. Further, the measure of hunger-coping economic tradeoffs could be more complex. The data did not elucidate the financial magnitude of the shortfalls in food and other basic needs. Additionally, whether those basic needs were partially or fully met could not be determined.

Although hunger-coping economic tradeoffs may be smaller financial constraints than medical debt, the tradeoffs could also be persistent or variable financial burdens over time. Future work that measures the persistence and variability of hunger-coping economic tradeoffs longitudinally could provide insight into which short-term financial strategies persist over time to become long-term strategies. Further, the cross-sectional nature of the data precludes detailed examination of mechanisms underlying the relationship between medical debt and hunger coping economic tradeoffs. Qualitative studies could provide insight into potential mechanisms and an understanding of how individuals decide between paying for medical bills and meeting basic needs. This type of information can inform future data collections and the design of targeted interventions that strengthen both financial and nutritional resilience. Nonetheless, our results support the need to address diverse hunger-coping tradeoffs in this community.

## 5. Conclusions

The findings underscore the emergence of medical debt as a salient correlate of food and basic needs challenges among households. Specifically, recipients with medical debt were more likely to employ multiple short-term strategies referred to as hunger-coping economic tradeoffs to meet basic needs. Financial literacy courses may help reduce hunger-coping economic tradeoffs. Food distribution sites may provide an excellent opportunity to provide collaborative interventions to address medical debt in a non-healthcare setting, in addition to providing food and resources to address other basic needs.

## Figures and Tables

**Table 1:** Descriptive Statistics of the Full Analytic Sample and by Medical Debt Status.

	Full Analytic Sample (N = 999)				Medical Debt (n = 405)				No Medical Debt (n = 594)				*p*-Value ^a^
	#	% or Mean	SD	Range	#	% or Mean	SD	Range	#	% or Mean	SD	Range	
Hunger-Coping Economic Tradeoffs													
Childcare vs. Food	138	13.81%			74	18.27%			64	10.77%			*p* < 0.01
Utilities vs. Food	467	46.75%			231	57.04%			236	39.73%			*p* < 0.001
Rent/Mortgage vs. Food	406	40.64%			207	51.11%			199	33.50%			*p* < 0.001
Transportation vs. Food	419	41.94%			230	56.79%			189	31.82%			*p* < 0.001
Education vs. Food	137	13.71%			80	19.75%			57	9.60%			*p* < 0.001
Medical Debt Status													
Medical Debt	405	40.54%			405	100.00%			--	--			
No Medical Debt	594	59.46%			--	--			594	100.00%			
Sociodemographic Characteristics													
Age, years		55.41	14.30	19–92		55.68	13.57	21–92		55.22	14.78	19–85	*p* < 0.001
Sex													
Male	231	23.12%			88	21.73%			143	24.07%			
Female	768	76.88%			317	78.27%			451	75.93%			
Race/Ethnicity													
Black	358	35.84%			167	41.23%			191	32.15%			*p* < 0.01
Hispanic	509	50.95%			164	40.49%			345	58.08%			*p* < 0.001
White	78	7.81%			52	12.84%			26	4.38%			*p* < 0.001
Other Race	54	5.41%			22	5.43%			32	5.39%			
Marital Status													
Married	479	47.95%			194	47.90%			285	47.98%			
Single	520	52.05%			211	52.10%			309	52.02%			
Education													
<HS diploma	347	34.73%			117	28.89%			230	38.72%			*p* < 0.01
HS diploma	280	28.03%			115	28.40%			165	27.78%			
>HS diploma	372	37.24%			173	42.72%			199	33.50%			*p* < 0.01
Income													
$0–$10,000	242	24.22%			84	20.74%			158	26.60%			*p* < 0.05
$10,001–$15,000	223	22.32%			93	22.96%			130	21.89%			
$15,001–$25,000	292	29.23%			129	31.85%			163	27.44%			
≥$25,001	242	24.22%			99	24.44%			143	24.07%			
Household Employment													
Full-time	302	30.23%			124	30.62%			178	29.97%			
Part-time	205	20.52%			81	20.00%			124	20.88%			
Not Currently Employed	492	49.25%			200	49.38%			292	49.16%			
Health Insurance													
Private	202	20.22%			98	24.20%			104	17.51%			*p* < 0.05
Medicaid	403	40.34%			156	38.52%			247	41.58%			
Medicare	168	16.82%			67	16.54%			101	17.00%			
Uninsured	226	22.62%			84	20.74%			142	23.91%			
SNAP	300	30.03%			129	31.85%			171	28.79%			
# of Household Members		3.69	1.89	1–8		3.92	1.94	1–8		3.54	1.84	1–8	*p* < 0.01
Language of Survey													
Spanish	371	37.14%			113	27.90%			258	43.43%			*p* < 0.001
English	628	62.86%			292	72.10%			336	56.57%			
Survey Location													
Atlanta	355	35.54%			174	42.96%			181	30.47%			*p* < 0.001
Houston	644	64.46%			231	57.04%			413	69.53%			

^a^ Bivariate analyses were conducted to compare hunger-coping economic tradeoffs and sociodemographic characteristics by the presence of medical debt. SD = standard deviation; HS = high school; SNAP = Supplemental Nutrition Assistance Program.

**Table 2:** Covariate-Adjusted Logistic Regression Models for Medical Debt as a Predictor of Hunger-Coping Economic Tradeoffs (N = 999). adjusted Odds Ratio [95% CI].

	Hunger-Coping Economic Tradeoffs									
	Food or Childcare		Food or Utilities		Food or Rent/Mortgage		Food or Transportation		Food or Education	
Medical Debt	1.92 ***	[1.37, 2.69]	2.05 ***	[1.59, 2.64]	2.34 ***	[1.75, 3.14]	2.81 ***	[2.05, 3.84]	2.18 ***	[1.42, 3.34]
Sociodemographic Characteristics										
Age	0.97 ***	[0.95, 0.98]	0.99 *	[0.98, 1.00]	0.98 ***	[0.97, 0.99]	0.98 **	[0.97, 0.99]	0.97 **	[0.96, 0.99]
Sex										
Male	1.00	--	1.00	--	1.00	--	1.00	--	1.00	--
Female	1.06	[0.69, 1.62]	1.53 *	[1.06, 2.21]	0.76	[0.53, 1.08]	0.79	[0.57, 1.11]	1.22	[0.75, 1.98]
Race/Ethnicity										
Black	1.00	--	1.00	--	1.00	--	1.00	--	1.00	--
Hispanic	0.65	[0.31, 1.37]	1.05	[0.68, 1.61]	0.95	[0.56, 1.61]	0.68	[0.40, 1.16]	0.53	[0.24, 1.19]
White	0.35 *	[0.13, 0.94]	0.86	[0.57, 1.28]	0.78	[0.49, 1.22]	0.71	[0.46, 1.10]	0.78	[0.40, 1.52]
Other Race	2.04	[0.88, 4.75]	1.17	[0.67, 2.07]	1.13	[0.54, 2.38]	0.68	[0.35, 1.34]	1.11	[0.53, 2.32]
Marital Status										
Married	0.74	[0.46, 1.17]	0.96	[0.71, 1.29]	0.79	[0.56, 1.10]	0.78 *	[0.62, 0.99]	0.83	[0.59, 1.17]
Single	1.00	--	1.00	--	1.00	--	1.00	--	1.00	--
Education										
<HS diploma	1.00	--	1.00	--	1.00	--	1.00	--	1.00	--
HS diploma	0.82	[0.54, 1.26]	0.88	[0.64, 1.20]	0.76	[0.50, 1.13]	0.99	[0.71, 1.38]	0.60	[0.35, 1.02]
>HS diploma	0.88	[0.54, 1.43]	0.88	[0.60, 1.29]	0.83	[0.57, 1.20]	0.87	[0.61, 1.24]	1.04	[0.73, 1.48]
Income										
$0–$10,000	1.00	--	1.00	--	1.00	--	1.00	--	1.00	--
$10,001–$15,000	0.75	[0.44, 1.29]	1.16	[0.90, 1.51]	1.05	[0.77, 1.42]	0.72 *	[0.54, 0.97]	0.91	[0.62, 1.35]
$15,001–$25,000	0.82	[0.45, 1.49]	0.88	[0.57, 1.36]	0.95	[0.63, 1.43]	0.83	[0.55, 1.27]	1.06	[0.55, 2.02]
≥$25,001	0.63	[0.27, 1.52]	0.60 **	[0.41, 0.88]	0.54 ***	[0.40, 0.72]	0.70	[0.45, 1.10]	0.84	[0.39, 1.81]
Household Employment										
Full-time	0.87	[0.53, 1.42]	1.29	[0.92, 1.81]	1.64 *	[1.08, 2.51]	1.06	[0.66, 1.70]	1.20	[0.74, 1.93]
Part-time	0.74	[0.46, 1.19]	1.21	[0.82, 1.80]	1.39	[0.95, 2.03]	1.32	[0.91, 1.91]	0.96	[0.52, 1.75]
Not Currently Employed	1.00	--	1.00	--	1.00	--	1.00	--	1.00	--
Health Insurance										
Private	1.00	--	1.00	--	1.00	--	1.00	--	1.00	--
Medicare	0.85	[0.42, 1.74]	1.34	[0.94, 1.91]	1.29	[0.93, 1.79]	1.29	[0.85, 1.94]	0.99	[0.59, 1.66]
Uninsured	0.92	[0.46, 1.85]	1.21	[0.83, 1.75]	1.07	[0.69, 1.65]	1.54	[0.96, 2.47]	0.77	[0.40, 1.46]
Medicaid	1.44	[0.82, 2.54]	1.63	[0.98, 2.70]	1.89 **	[1.27, 2.80]	1.63 *	[1.10, 2.42]	0.99	[0.59, 1.66]
SNAP	1.31	[0.78, 2.20]	0.92	[0.65, 1.30]	0.99	[0.67, 1.46]	0.92	[0.71, 1.20]	0.82	[0.50, 1.33]
# of Household Members	1.25 ***	[1.11, 1.41]	1.04	[0.99, 1.09]	1.08	[0.99, 1.17]	1.10 **	[1.04, 1.18]	1.18 *	[1.03, 1.35]
Language of Survey										
Spanish	1.75	[0.87, 3.54]	0.80	[0.47, 1.37]	1.18	[0.70, 1.97]	0.78	[0.42, 1.44]	1.25	[0.64, 2.44]
English	1.00	--	1.00	--	1.00	--	1.00	--	1.00	--
Survey Location										
Atlanta	1.76	[0.89, 3.47]	1.17	[0.80, 1.71]	1.27	[0.71, 2.28]	1.15	[0.81, 1.64]	1.09	[0.63, 1.89]
Houston	1.00	--	1.00	--	1.00	--	1.00	--	1.00	--

* *p* < 0.05, ** *p* < 0.01, *** *p* < 0.001. HS = high school; SNAP = Supplemental Nutrition Assistance Program.

## Data Availability

The data used in this study are confidential.
